# Rational design of a heterotrimeric G protein α subunit with artificial inhibitor sensitivity

**DOI:** 10.1074/jbc.RA118.007250

**Published:** 2019-02-11

**Authors:** Davide Malfacini, Julian Patt, Suvi Annala, Kasper Harpsøe, Funda Eryilmaz, Raphael Reher, Max Crüsemann, Wiebke Hanke, Hang Zhang, Daniel Tietze, David E. Gloriam, Hans Bräuner-Osborne, Kristian Strømgaard, Gabriele M. König, Asuka Inoue, Jesus Gomeza, Evi Kostenis

**Affiliations:** From the ‡ Molecular, Cellular and Pharmacobiology Section, Institute for Pharmaceutical Biology, and; ¶ Institute for Pharmaceutical Biology, University of Bonn, Nussallee 6, 53115 Bonn, Germany,; § Department of Drug Design and Pharmacology, University of Copenhagen, Universitetsparken 2, 2100 Copenhagen, Denmark,; ‖ Eduard Zintl Institute of Inorganic and Physical Chemistry, Technische Universität Darmstadt, 64287 Darmstadt, Germany, and; ** Graduate School of Pharmaceutical Sciences, Tohoku University, 6-3 Aoba, Aramaki, Aoba-ku, Sendai, Miyagi 980-8578, Japan

**Keywords:** G protein, G protein-coupled receptor (GPCR), signal transduction, pharmacology, CRISPR/Cas, DMR, FR900359, label-free, YM-254890

## Abstract

Transmembrane signals initiated by a range of extracellular stimuli converge on members of the Gq family of heterotrimeric G proteins, which relay these signals in target cells. Gq family G proteins comprise Gq, G11, G14, and G16, which upon activation mediate their cellular effects via inositol lipid–dependent and –independent signaling to control fundamental processes in mammalian physiology. To date, highly specific inhibition of Gq/11/14 signaling can be achieved only with FR900359 (FR) and YM-254890 (YM), two naturally occurring cyclic depsipeptides. To further development of FR or YM mimics for other Gα subunits, we here set out to rationally design Gα16 proteins with artificial FR/YM sensitivity by introducing an engineered depsipeptide-binding site. Thereby we permit control of G16 function through ligands that are inactive on the WT protein. Using CRISPR/Cas9-generated Gαq/Gα11-null cells and loss- and gain-of-function mutagenesis along with label-free whole-cell biosensing, we determined the molecular coordinates for FR/YM inhibition of Gq and transplanted these to FR/YM-insensitive G16. Intriguingly, despite having close structural similarity, FR and YM yielded biologically distinct activities: it was more difficult to perturb Gq inhibition by FR and easier to install FR inhibition onto G16 than perturb or install inhibition with YM. A unique hydrophobic network utilized by FR accounted for these unexpected discrepancies. Our results suggest that non-Gq/11/14 proteins should be amenable to inhibition by FR scaffold–based inhibitors, provided that these inhibitors mimic the interaction of FR with Gα proteins harboring engineered FR-binding sites.

## Introduction

GTP/GDP exchange and the intrinsic GTPase activity of GTP-binding proteins constitute widespread regulatory mechanisms in cells. These are utilized by heterotrimeric αβγ G proteins, downstream effectors of G protein–coupled receptors (GPCRs),^6^ to directly or indirectly regulate numerous physiological processes in mammals (1–4). G proteins are grouped into four major families (Gs, Gi/o, Gq/11, and G12/13) on the basis of α subunit homology and function (3, 5–7). Common to all G proteins is a highly conserved molecular architecture and the mechanism they use to allow cells to respond to extracellular cues (8–12).

Previous X-ray crystallographic analysis revealed that G proteins from different organisms and subclasses share nearly identical structural features (9–16). They are composed of two domains, a GTPase “Ras-like” domain that is conserved in all members of the GTPase superfamily and is homologous to the monomeric GTPase Ras and an all-α-helical domain that is unique to heterotrimeric G proteins (17, 18). Both domains are held together by two flexible linkers and tightly sandwich the guanine nucleotide (19, 20). Separation of Ras and the helical domain to facilitate GDP/GTP exchange and thereby initiate cellular signaling may occur spontaneously (21–24) or in response to internal structural rearrangement triggered by activated GPCRs that act as guanine nucleotide exchange factors (4, 25–27). At steady state, signaling output is balanced by the competing actions of GPCRs, which accelerate GTP binding, and GTPase-activating proteins, which accelerate GTP hydrolysis (28). In this way, Gα proteins function as nucleotide state–dependent molecular switches that are critical to defining the duration of G protein signaling.

**Figure 1.**
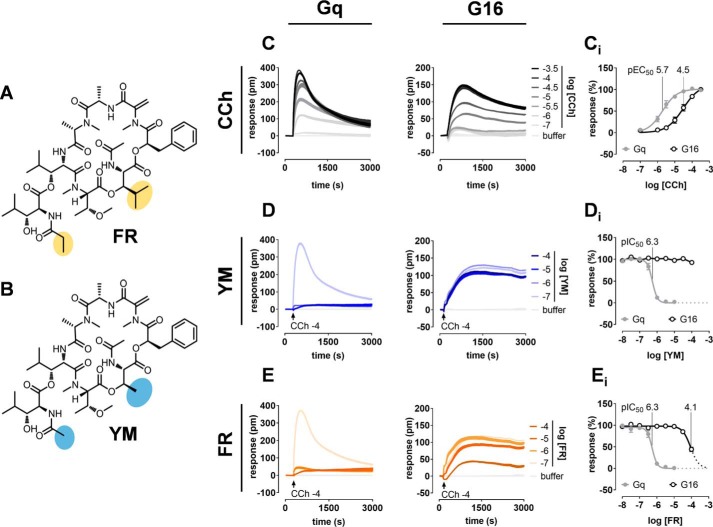
**FR and YM differentially inhibit signaling of Gq family members Gαq and Gα16.** Chemical structures of FR (*A*) and YM (*B*) with differing moieties highlighted in *yellow* and *blue*, respectively. *C*, concentration-dependent activation profiles of CCh in HEK293 Gαq/Gα11-null cells transfected to express WT Gαq or Gα16 using label-free DMR biosensing. *C_i_*, concentration-effect curves of the traces depicted in *C. D* and *E*, concentration-dependent inhibition of cell responses induced with CCh at its EC_80_ by YM (*D*) and FR (*E*) in cells expressing Gαq or Gα16. *D_i_* and *E_i_*, concentration-inhibition relationships for the traces shown in *D* and *E*. DMR recordings are representative (mean + S.E.) of at least four independent biological replicates conducted in triplicate (*C–E*), whereas concentration-effect relationships are means ± S.E. from at least four independent biological replicates. *pm*, wavelength shift in picometer.

Although this form of signal transduction is basic to the mechanisms that cells have evolved for responding to hormones, neurotransmitters, and a variety of other ligands, pharmacological control of G protein function is by far more difficult to achieve (29, 30). Indeed, despite discovery of heterotrimeric G proteins about 30 years ago, very few pharmacological agents allow precision control of the G protein cycle. Among these are the two bacterial toxins, pertussis toxin and cholera toxin, which act via covalent modification of the Gα subunits Gαi and Gαs to ablate Gi and mask Gs coupling, respectively (31, 32). However, noncovalent control of G protein function in a subfamily-specific manner has so far only been accomplished for Gq family proteins. One such notable Gq-inhibiting agent is FR900359 (FR; see Fig. 1*A*), a plant-derived cyclic depsipeptide with exceptional selectivity for Gq, G11, and G14 over all other G proteins. Mechanistically, FR interdicts Gq activity via inhibition of GDP release and was therefore classified by us as a guanine nucleotide dissociation inhibitor (33). Because FR is active in intact cells (33–37), on the level of isolated organs (33, 38, 39), and in the living organism (36, 38, 40), it has been used widely to probe the biological consequences that arise from specific inhibition of Gq signaling cascades *in vitro* and *in vivo* (33, 36, 38). How FR achieves this specific inhibition at the molecular level is presently unknown.

Therefore, the first goal of our study was to map the site of FR action within Gαq by mutational analysis. This was guided by and based on the cocrystal structure of YM-254890 (YM; see Fig. 1*B*), a structurally closely related Gq inhibitor, in complex with a Gi/q chimeric protein, which previously provided a framework for understanding YM's action in great molecular detail (19). Unexpectedly, we found that FR is distinct from YM in that shared but divergent amino acid networks within a common binding site account for its biological effects. Nevertheless, this common site is of interest for two reasons: it is sufficiently conserved to imply that other Gα subunits may be inhibited by similar mechanisms but sufficiently diverse to posit that FR or YM analogs may be developed with altered Gα specificity profiles. However, to date and despite intense efforts (41–47), no single FR or YM analog inhibits G proteins apart from Gq, G11, and G14 (41, 44, 46–48), raising the possibility that rational design of such molecules may be more challenging than generally anticipated (1, 19, 33, 45).

Therefore, rather than engineering additional novel analogs, our second goal was to engineer novel Gα proteins with artificial sensitivity toward FR and YM. In the study described herein, we chose Gα16, which is not naturally regulated by FR, for reconstruction of functional inhibitor sites. Although the ultimate goal of our proof-of-concept study is to facilitate discovery of FR scaffold–based inhibitors with altered Gα specificities, Gα proteins with engineered depsipeptide-binding sites may be considered a first step along this path to support rational design of such molecules.

## Results

### Differential inhibition of Gq family proteins by FR and YM

In-depth insight into inhibitor topology on WT Gαq is a prerequisite for reconstruction of functional FR/YM sites into Gα proteins that are naturally not inhibitor-regulated. Although select interaction points between YM and Gαq have been identified previously by cocrystal and mutational analyses (19), it is elusive at present whether Gαq proteins can be designed that maintain catalytic function but are mutationally resistant to the inhibitor. Because FR and YM inhibit Gq, G11, and G14 but not G16, the closest FR-insensitive relative, we reasoned that a catalytically active depsipeptide-resistant Gq should result from switching the relevant Gαq residues to their counterpart sequences in Gα16 provided that G16 is inert to both inhibitors. Therefore, we investigated a possible direct inhibition by FR and YM of G16 using real-time live-cell phenotypic biosensor assays based on dynamic mass redistribution (DMR) in CRISPR/Cas9 genome-edited HEK293 cells deficient in Gαq and Gα11 (hereafter “Gαq/Gα11-null” cells). This cellular background allows analysis of Gq family proteins without the confounding variable of endogenously expressed Gαq and Gα11. As expected, Gαq/Gα11-null cells were unresponsive to carbachol (CCh), which activates Gq-sensitive endogenous muscarinic M3 receptors (Fig. S1), but displayed robust and concentration-dependent activity profiles upon re-expression of WT Gαq or Gα16 (Fig. 1*C*). In agreement with its reported selectivity profile, YM potently inhibited Gq but was completely inactive at G16 (Fig. 1*D*). FR, in contrast, blunted Gq activation with potency equivalent to that of YM and, additionally, G16 at the highest applied concentrations (Fig. 1*E*). Inhibition of cell function at high FR concentrations did not result from off-target activity but was specific to G16 because FR (i) did not produce any DMR when applied alone (Fig. S2*A*) and (ii) did not diminish signals evoked with epidermal growth factor as a non-Gq stimulus (Fig. S2*B*). Thus, despite their structural similarity, FR is distinct from YM in that it does display some residual interaction with G16, a feature that is also recapitulated in the more traditional inositol monophosphate (IP_1_) accumulation assay (Fig. S3) and that may impact gain- and loss-of-function mutagenesis.

### Key interaction points for YM inhibition of Gq affect FR to a lesser extent

**Figure 2.**
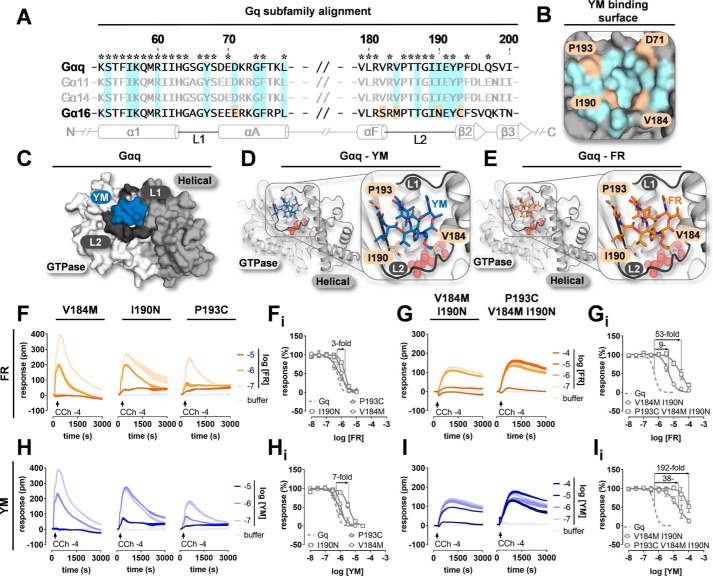
**Key interaction points for YM inhibition of Gq affect FR to a lesser extent.** *A*, amino acid sequence alignments and secondary structure features of Gq family α subunits. Secondary structure assignments (α-helices, β-strands, and linker regions) and ruler numbering are derived from Gαi/q (PDB code 3AH8). Conserved residues are marked with *asterisks*. Gαq residues implicated both directly and indirectly in YM interaction are highlighted in *light blue*. Gα16 residues that differ from Gαq in either direct YM-binding regions or adjacent areas are color-coded *ochre. B*, interaction surface of YM on the Gαq tertiary structure including Asp-71; the color code of the sequence alignment is kept to visualize direct YM–Gαq contacts (*light blue*) and sequence deviations from Gα16 at equivalent locations (*ochre*). *C*, surface representation of YM-bound Gαi/q tertiary structure (PDB code 3AH8) composed of the GTPase and helical domain that are connected by linker 1 (*L1*) and linker 2 (*L2*; =switch I), respectively. *D* and *E*, features of YM (*D*) and FR (*E*) interaction with Gαq as overall and close-up views. Residues previously defined as important for YM–Gαq interaction are shown as *sticks* and colored *beige*. FR was modeled into the Gαq-binding site assuming equivalent anchor points as compared with YM; GDP is shown as *spheres. F–I*, inhibition by FR (*F* and *G*) and YM (*H* and *I*) of G protein–dependent whole-cell activation profiles in CRISPR/Cas9 Gαq/Gα11-null cells transfected to express the indicated Gαq single (*F* and *H*) and double and triple (*G* and *I*) mutants. G proteins were activated via endogenous M3 receptors upon challenge with CCh at its EC_80_. Shown are representative whole-cell recordings along with concentration-inhibition curves for each individual mutant (*F_i_–I_i_*). -Fold shifts above the curves denote the loss of inhibitor potency for selected mutants *versus* WT Gαq. All DMR traces depict means of three technical replicates. Concentration-inhibition curves are means ± S.E. from at least three independent biological replicates. *pm*, wavelength shift in picometer.

We next explored key sites for FR inhibition of Gq by targeted mutagenesis. To this end, we initially replaced Gαq residues previously reported to engage in direct interactions with YM by the matching Gα16 counterparts: V184M, I190N, and P193C (19). In fact, of all 17 amino acids with side chains within 5 Å of YM, these three are completely conserved only in YM-sensitive Gαq/11/14, suggesting that they act as key specificity determinants (Fig. 2, *A* and *B*). They line the linker 2 region, also known as Switch I, which together with linker 1 provides the interdomain cleft for occupancy by YM to stabilize Gq in its GDP-bound form (Fig. 2, *C* and *D*). We predicted FR to engage Gαq the same way as YM, and as anticipated, modeled FR recapitulated this interaction (Fig. 2*E*). Unexpectedly, Gq inhibition assays showed that FR sensitivity of all three mutants was hardly affected (Fig. 2*F*). However, when single mutants were combined to double and triple mutants, FR sensitivity was markedly reduced by almost 2 orders of magnitude (Fig. 2*G*). These data corroborate the notion that linker 2/Switch I also ensures specificity of FR action but is not sufficient to explain selectivity entirely. A similar picture emerged from mutational analysis of YM with the only difference being that activity loss of YM was always greater than that of FR for individual mutants (Fig. 2, *H* and *I*).

### Shared and divergent amino acid networks explain differential inhibition of Gq family members by FR and YM

**Figure 3.**
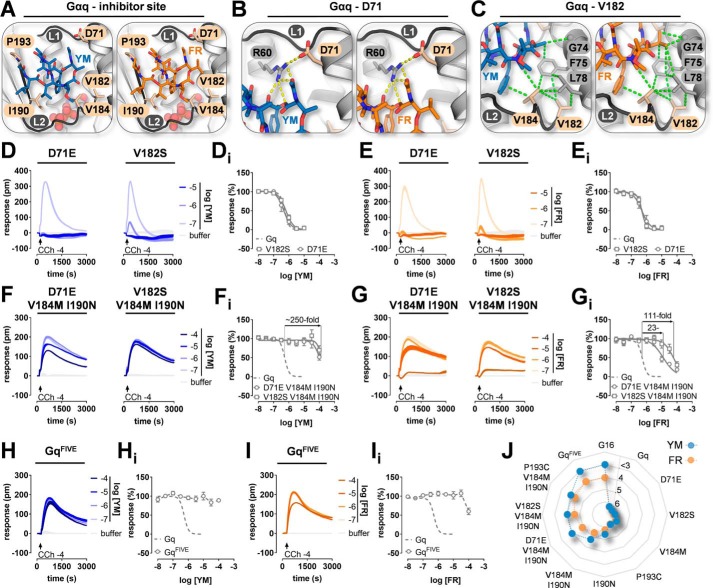
**Fewer amino acid changes are required to achieve mutational resistance of Gq to YM.** *A*, depsipeptide-binding site of YM- and FR-bound Gαq. Residues directly interacting with YM and FR (Val-184, Ile-190, and Pro-193) or involved in precise positioning of inhibitors at their target site (Asp-71 and Val-182) are shown in *sticks* and colored *beige*; GDP is shown as *spheres. B*, zoomed-in view into the Gαq Asp-71–Arg-60 salt bridge (*yellow stippled line*, Glu-74–Arg-63 in Gα16) that stabilizes YM– and FR–linker 1 interaction. Note that Asp-71 does not form specific contacts to YM and FR. *C*, zoomed-in view into hydrophobic interactions (*green stippled lines*) formed between YM or FR and the hydrophobic cluster in the Gαq interdomain cleft with relevant residues named and/or shown as *sticks* in *beige*. Note that FR produces more hydrophobic interactions as compared with YM. *D–I*, DMR analysis of Gαq loss-of-function mutants in CRISPR/Cas9 Gαq/Gα11-null cells transfected to express the indicated constructs. Data show concentration-dependent inhibition of G protein–mediated whole-cell activation profiles by YM (*blue*) and FR (*orange*) in cells harboring single (*D* and *E*), triple (*F* and *G*), and quintuple Gαq mutants (*H* and *I*). Shown are representative traces (*D–I*; mean + S.E. of technical triplicates) and concentration-inhibition relations that are means ± S.E. from at least three independent biological replicates (*D_i_–I_i_*). -Fold shifts above selected curves indicate loss of inhibitor sensitivity for individual mutants *versus* WT Gαq. *pm*, wavelength shift in picometer. *J*, pharmacological profiles of G protein inhibition that distinguish FR from YM. Plotted are the inhibitory potencies (pIC_50_) of FR and YM for all Gαq loss-of-function constructs with Gq and G16 WT for comparison. Activity of YM on Gq^FIVE^ and G16 is denoted as arbitrarily low (pIC_50_ < 3). This radial multiaxis plot provides a closed polygonal profile for each inhibitor, illustrating that YM becomes inactive with fewer amino acid changes.

Because none of our mutant proteins were inhibitor-resistant, we searched for additional residues that, when mutated, may incur activity loss for the two depsipeptides. We focused our attention on residues that (i) differed between Gαq and Gα16, (ii) were not addressed previously by mutagenesis, and (iii) were located on both linkers, including adjacent regions, to either directly interact with the depsipeptides (if side-chain or Gα atoms were within 5 Å to YM) or to be close enough to assist in stabilizing the inhibitor–protein interactions. Aspartate 71 (Asp-71) and valine 182 (Val-182) fulfilled these criteria (Fig. 3*A*; see also Fig. 2*A*): Asp-71 is part of an aspartic acid–arginine salt bridge that likely provides the interface between the depsipeptides and linker 1 by stabilizing and maintaining the inhibitor–linker 1 spacing (Fig. 3*B*). Because accurate placement of salt-bridging residues in proteins is crucial for intra- and intermolecular recognition (49–51), we reasoned that exchange of aspartate for glutamic acid, the matching Gα16 counterpart, may impact inhibitor sensitivity in a negative way. Likewise, replacement of the hydrophobic valine by the corresponding polar serine (V182S) may destabilize a hydrophobic network (Phe-75, Leu-78, Val-182, Val-184, and Ile-190) that is essential for YM and likely also for FR (Fig. 3*C*). Gq inhibition assays showed that individual substitutions were without effect on inhibitor function (Fig. 3, *D* and *E*), whereas combined replacement of either D71E or V182S within the V184M/I190N double mutant context essentially abolished YM sensitivity and severely compromised that of FR (Fig. 3, *F* and *G*). Replacement of all five diverging residues (Gq^FIVE^) was required and sufficient to attenuate FR action to the same extent as observed on WT G16. In fact, Gq^FIVE^ recapitulated the pharmacological profiles observed for FR and YM on native G16 in both DMR (compare Fig. 3, *H* and *I*, with Fig. 1, *D* and *E*) and IP_1_ accumulation assays (Fig. S4). For all Gαq loss-of-function mutants, potency measures for activation by CCh were similar to that of Gq WT (Fig. S5 and Table S1) with signaling amplitudes well correlated to cellular abundance (Fig. S6).

Together, our loss-of-function mutagenesis revealed two recurring themes: (i) inhibitors were largely unaffected by single point mutations (at best 7-fold for YM at I190N), and (ii) YM was less tolerant to substitutions as compared with FR, *i.e.* became inactive by fewer changes (Fig. 3*J* and Table S1). From these data, we concluded that a network of hydrophobic interactions rather than individual anchor points is essential for tightening the ligands to their target site. FR, which is more hydrophobic than YM (Figs. 1, *A* and *B*, and 3*C*), is more tolerant to loss of hydrophobic interactions and may, therefore, be more difficult to detach.

### Quantitative reconstruction of functional FR and YM sites within Gα16

**Figure 4.**
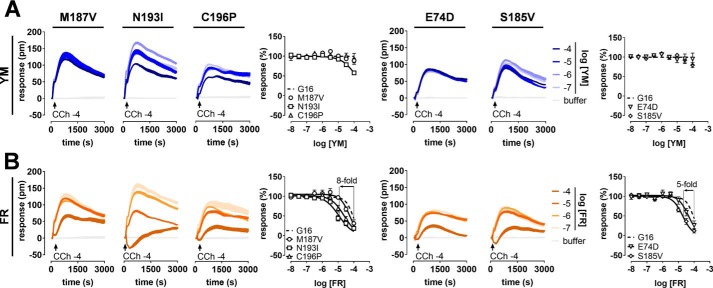
**Single gain-of-function mutants measurably support G16 inhibition by FR but not YM.** CRISPR/Cas9 Gαq/Gα11-null cells ectopically expressing the indicated Gα16 gain-of-function mutants were stimulated with CCh at its EC_80_ to enable quantification of inhibitory profiles for YM (*A*) and FR (*B*). This set of DMR recordings (technical triplicates) is representative of at least three additional experiments; concentration-inhibition profiles for each mutant are the means ± S.E. of at least three independent biological replicates .-Fold increases of FR inhibitory potency are indicated for selected mutants. *pm*, wavelength shift in picometer.

**Figure 5.**
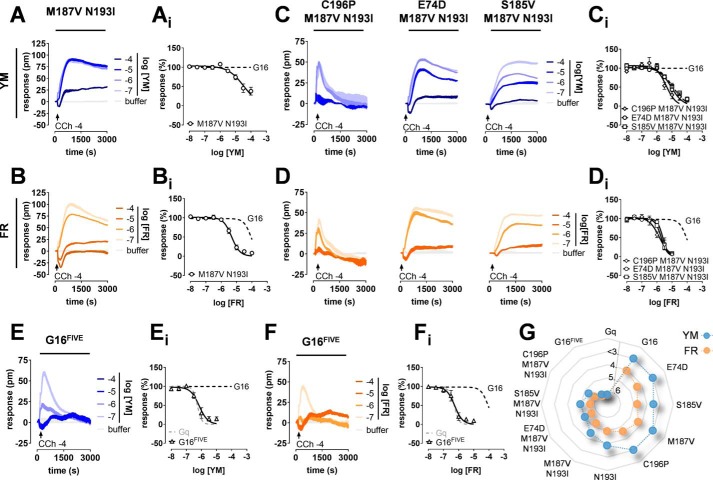
**Quantitative reconstruction of functional FR and YM sites within Gα16.** *A–F*, Gα16 sensitization toward inhibition by YM (*blue* traces) and FR (*orange* traces) is achieved by gradual build-up of inhibitor sites using double (*A* and *B*), triple (*C* and *D*), and quintuple mutants (*E* and *F*). Shown are representative real-time recordings (*A–F*; technical triplicates) and corresponding concentration-effect relationships (*A_i_–F_i_*; means ± S.E. from at least three independent biological replicates) obtained by DMR biosensing in CRISPR/Cas9 cells ectopically expressing the indicated Gα variants. *pm*, wavelength shift in picometer. *G*, wheel chart summarizing the divergent pharmacological profiles of FR and YM. Plotted are the inhibitory potencies (pIC_50_) of both inhibitors for all Gα16 gain-of-function mutants with Gq and G16 WT included as comparators. Lack of detectable YM inhibition is denoted as arbitrarily low activity (pIC_50_ < 3). The closed polygonal profiles indicate that inhibition of G16 by FR was more readily introduced than by YM.

Based on our loss-of-function mutagenesis, transplantation of an entire hydrophobic network including multiple interactions rather than individual amino acid replacements is likely required to install FR and YM sensitivity onto G16. Indeed, exchange of each of the five diverging amino acids by the matching Gαq counterparts had little to no effect on G16 inhibition by YM (Fig. 4*A*). Inhibition by FR, in contrast, was measurably improved by each individual substitution (Fig. 4*B*). We then gradually built up the inhibitor–Gαq interface using double (Fig. 5, *A* and *B*), triple (Fig. 5, *C* and *D*), and quintuple mutants (Fig. 5, *E* and *F*; see Figs. S7 and S8 for validation of function and expression of Gα16 mutants). Of these, G16^FIVE^, the quintuple mutant with combined replacement of all divergent residues, conferred the largest degree of G16 inhibition for FR and YM in both DMR (Fig. 5, *E* and *F*) and IP_1_ accumulation assays (Fig. S9). Although depsipeptide pharmacology on G16^FIVE^ was almost superimposable with that on Gq WT (compare Fig. 5, *E* and *F*, with Fig. 1, *D* and *E*), inhibition of G16 by FR was more readily introduced than by YM (Fig. 5*G* and Table S2).

### Unique hydrophobic interactions account for the divergent pharmacological profiles of FR and YM

**Figure 6.**
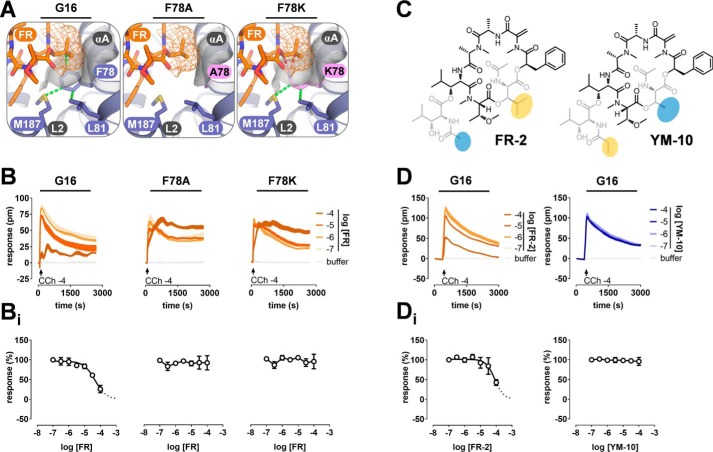
**Hydrophobic Phe-78 buried in the interdomain cleft accounts for inhibition of G16 by FR.** *A*, homology model of FR-bound Gα16 based on YM-bound Gαi/q (PDB 3AH8) with *orange mesh* and *gray surface* representing the vdW (van der Waals) surface of FR and Gα16, respectively. FR, via its *N*-acetyl-hydroxyleucine side chain (the “isopropyl moiety”), engages in hydrophobic interactions with Phe-78 (*green stippled lines*) in Gα16 WT. This interaction cannot take place in F78A, as represented by the significant reduction in surface complementarity, or in F78K, where repulsive forces (*red stippled lines*) between Lys-78 and FR likely make favorable interactions impossible. *B*, CRISPR/Cas9 Gαq/Gα11-null cells transfected to express Gα16 or the indicated mutant isoforms were pretreated with FR at the indicated concentrations prior to stimulation with CCh at its EC_80_. High concentrations of FR inhibit G16-mediated cell responses but not those evoked by F78A and F78K mutants. *C*, chemical structures of the FR and YM hybrid molecules FR-2 (43) and YM-10 (45). The naturally occurring FR-2 retains the *N*-acetyl-hydroxyleucine building block of FR (*yellow* marking) along with the ester-linked side chain of YM (*N*-acetyl-hydroxyleucine; *blue* marking). YM-10 contains the *N*-acetyl-threonine building block of YM (*blue* marking) but the ester-linked side chain of FR, which is composed of an *N*-propionyl-hydroxyleucine (*yellow* marking). *D*, effects of FR-2 and YM-10 on CCh-mediated G16 activation in CRISPR/Cas9 Gαq/Gα11-null cells. Data shown in *B* and *D* are representative real-time recordings (technical triplicates) along with concentration-inhibition relations (*B_i_* and *D_i_*) that depict means ± S.E. from three independent biological replicates. If not shown, *error bars* lie within dimensions of the *symbols. pm*, wavelength shift in picometer.

**Figure 7.**
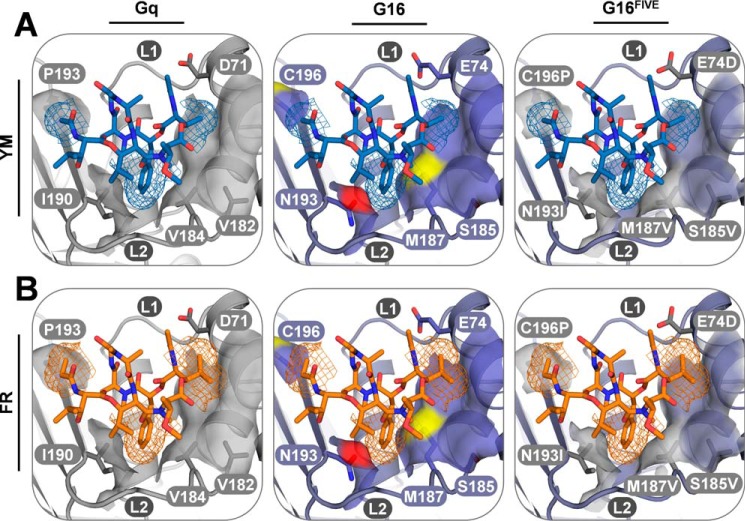
**Unique hydrophobic interactions account for the divergent pharmacological profiles of FR and YM.** YM (*blue*; *A*) and FR (*orange*; *B*) bound to Gαq plus homology models of Gα16 and Gα16^FIVE^ highlighting (*thin stick* representation) key residues that engage in direct interactions with both inhibitors or contribute indirectly via stabilization of hydrogen-bonding or hydrophobic interactions are shown. *Blue* and *orange mesh* represents the vdW surfaces of YM and FR, respectively, whereas *gray* (carbon) and *purple*/*red*/*yellow* (carbon/oxygen/sulfur) *transparent surfaces* illustrate the vdW surface of Gαq-conserved and Gα16-specific residues, respectively. Due to the ethyl and isopropyl *versus* methyl moieties, FR *versus* YM displays significantly larger vdW contact surface complementarity to Pro-193 and the hydrophobic cluster (including positions Val-182/Ser-185 and Val-184/Met-187) in the binding site of all three Gα proteins. These additional hydrophobic contacts partly compensate for the weakened hydrophobic cluster and overall less hydrophobic nature of the binding site in Gα16 (Ser-185, Met-187, Asn-193, and Cys-196), (i) making FR binding to, and inhibition of, Gq less vulnerable to mutations and (ii) explaining the FR *versus* YM inhibition of WT G16 at high concentrations.

We reasoned that extent and magnitude of hydrophobic interactions may account for this phenomenon and that FR may be superior in harnessing the benefit of such interactions. In line with this prediction, FR inhibition was abolished in Gα16 mutants designed to diminish (F78A) or disrupt (F78K) key hydrophobic contacts between FR and the side chain of Phe-78, the Phe-75 counterpart and only remnant of the hydrophobic tetrad within the G16 interdomain cleft (Fig. 6, *A* and *B*; see Fig. S10 for validation of expression and function of both mutants). Likewise, only the naturally occurring FR-2 (43) but not the fully synthetic YM-10 (45) retained inhibition of G16 at high concentrations (Fig. 6, *C* and *D*). Thus, structural modifications on the ligand and the protein side corroborate the notion that Phe-78 is crucial for G16 anchorage of FR at high concentrations and likely also for the ease to install FR inhibition onto G16 with fewer mutations. More generally, the striking differences in the extent of hydrophobic interactions formed between the inhibitors and their protein targets likely explain the divergent pharmacological profiles of FR and YM (Fig. 7). Relative to Gq, G16 has significantly fewer hydrophobic residues than what would be needed to accommodate FR or YM. However, introduction of the hydrophobic cluster is required and sufficient to convert FR and YM into potent inhibitors of this naturally not FR/YM-regulated protein. Therefore, we anticipate that FR and YM mimics may employ this same basic mechanism of inhibition to silence function of FR/YM-insensitive Gα proteins.

## Discussion

FR and YM, two naturally occurring cyclic depsipeptides, are invaluable pharmacological tools for probing Gq-mediated cellular responses. Because of their specificity, they have become instrumental in defining and diagnosing the contribution of Gq proteins to complex biological processes *in vitro* and *ex vivo* (33–39, 52–59). FR and YM share a common mechanism of G protein inhibition: they act as guanine nucleotide dissociation inhibitors that preserve GDP-bound heterotrimers in their inactive state (19, 33). Although there is precedence for this mechanism of action (60), their site of action is unique. X-ray crystallographic evidence revealed that YM “dives” into a cleft between two interdomain linkers that connect the GTPase and the helical domain of Gα, which buries the bound nucleotide (19). Stabilization of these interdomain linkers directly accounts for suppression of GDP release by YM because the hinge motion that is required for movement of the helical domain away from the GTPase domain to facilitate nucleotide exchange and thereby initiate cellular signaling cannot take place. The mutagenesis results of our study suggest that FR operates via comparable mechanisms to achieve its specific blockade of Gq.

Although each Gα subunit preserves the interdomain cleft, FR and YM only stabilize Gαq, Gα11, and Gα14 in their GDP-bound forms (19, 33). The basis for this remarkable specificity likely lies in the nature of molecular recognition of YM and FR by their protein targets: hydrophobic interactions dominate the interface between the depsipeptides and Gαq at this interdomain cleft (Fig. 7). YM and even more so FR create an extensive hydrophobic interface that interacts with nonpolar amino acids of Gαq/11/14 (Phe-75, Leu-78, Val-184, and Ile-190; Gαq residue numbering), which account for a significant proportion of nonpolar contacts within the interdomain cleft. Equivalent interactions between FR or YM and the remaining Gα subunits, including Gα16, cannot be formed because their interdomain cleft is considerably less hydrophobic (Fig. 7). However, pharmacological control over G16 by FR scaffolds is achievable provided all relevant anchoring points are installed to mimic the interaction between FR and WT Gαq. Similarly, a fully functional FR site has recently been engineered into FR-insensitive Gαi (61). This was achieved by swapping a total of eight residues at a time of Gαi for the equivalent Gαq amino acids to enable effective allosteric inhibition of nucleotide exchange by FR (61). Although only two of the eight mutated residues were identical in G16^FIVE^ (Ile-190 and Pro-193; Gq numbering), these findings attest to the notion that the number of required amino acid switches for gain of FR inhibition likely correlates to the extent of sequence divergence within the FR-binding site.

It follows that the molecular architecture of the interdomain region confined by two flexible linkers offers unique opportunities for specific pharmacological targeting of Gα protein function with FR or YM mimics. However, to date, no single analog prepared by chemical synthesis or isolated from natural sources has shown activity on non-Gq/11/14 proteins (42, 46). Conceivably, design of FR or YM analogs with altered Gα specificities may be more challenging than generally anticipated (1, 19, 33, 45). This may be due, at least in part, to the fact that FR and YM are structurally complex molecules that likely require a network of residues for efficient engagement of their protein targets (42). Herein, we used site-directed mutagenesis and computational predictions to (i) define this network within Gαq and (ii) transplant it to Gα16, a Gq family member that is naturally not regulated by the inhibitors. By substituting Gαq residues with their counterpart Gα16 sequences and vice versa, we succeeded to completely swap pharmacology of one Gα subunit to that of the other for both depsipeptides. We noted that potency contributions of individual amino acids were not simply additive but synergistic (Fig. S11), indicating that alteration of one residue influences inhibitor activity at the others. This interdependence suggests that multiple simultaneous interactions are required for selective recognition of cognate depsipeptide ligands. Accordingly, we expect multiple simultaneous changes to be made to create FR or YM mimics that allow efficient engagement of non-Gq targets.

Precision pharmacological targeting of heterotrimeric G proteins in a Gα-specific manner still poses considerable challenges. Among all molecules interfering with G protein function, FR and YM are unique for their specific action on Gq family proteins. Apparently, only nature has overcome the major hurdles for development of Gα-specific inhibitors by evolving FR and YM for optimal interaction with Gq, G11, and G14. It will be intriguing to see whether depsipeptide mimics with altered Gα selectivity profiles may become accessible via semi- or total synthesis or via extension of nature's chemistry through combinatorial biosynthesis involving reconstitution and manipulation of biosynthetic enzymes in heterologous expression systems. Regardless, our here-designed depsipeptide-controlled Gα16 mutants provide (i) proof of principle that FR-insensitive Gα subtypes may be targeted by similar mechanisms and (ii) guidance for rational design of FR mimics that recapitulate the modeled interactions between FR and Gα16 proteins harboring engineered FR-binding sites.

## Conclusions

Hydrophobic interactions have long been recognized as major driving forces for macromolecular stability and complex formation (62–64). FR and YM, two naturally occurring cyclic depsipeptides, take advantage of such interactions to mediate their specific inhibition of Gq. In turn, absence of the hydrophobic residues at equivalent positions in other Gα subunits is consistent with the lack of FR/YM inhibition in these cases. We have shown in our study that the hydrophobic functional groups that discriminate FR from YM augment the contours of the binding surface beyond that which can be achieved with YM alone. We provided experimental evidence that hydrophobic interactions explain the residual activity of FR on G16 and posit their decisive importance to also account for the “superiority” of FR over YM in the gain- and loss-of-function mutants. Therefore, FR may be preferred over YM as privileged scaffold in the search for specific and potent modulators targeting G16.

## Experimental procedures

### Materials and reagents

Cell culture materials were purchased from Invitrogen. FR (previous commercial name UBO-QIC) and FR-2 were isolated and purified as described previously (33, 43). YM was obtained from Wako Chemicals GmbH (Neuss, Germany). The synthesis of YM-10 is detailed in Xiong *et al*. (45). Primary antibodies to detect Gα16, the human influenza hemagglutinin (HA) epitope tag (YPYDVPDYA), and β-actin were from Santa Cruz Biotechnology (Dallas, TX), Roche Applied Science, and BioLegend (San Diego, CA), respectively. The horseradish peroxidase (HRP)-conjugated secondary antibodies goat anti-mouse IgG and goat anti-rabbit IgG were from Sigma-Aldrich and Antibodies-online GmbH (Aachen, Germany), respectively. All other reagents were purchased from Sigma-Aldrich if not stated otherwise.

### Cell culture

Generation of genetically engineered HEK293 cells using CRISPR/Cas9 technology to knock out the subunits of Gαq and Gα11 (Gαq/Gα11-null cells) is described elsewhere (33). Genome-edited Gαq/Gα11-null cells were used to establish pooled clones stably expressing Gα16 WT and the mutant isoforms Gα16^E74D M187V N193I^, Gα16^S185V M187V N193I^, and Gα16^FIVE^ that were maintained under Zeocin^TM^ selection (200 μg/ml). Cells were maintained in Dulbecco's modified Eagle's medium supplemented with 10% (v/v) fetal calf serum, 100 units/ml penicillin, and 0.1 mg/ml streptomycin and kept in a humidified atmosphere with 5% CO_2_ at 37 °C.

### Site-directed mutagenesis and transfection

Mutations of the HA-tagged mouse Gαq cDNA and the human Gα16 cDNA in pcDNA3.1(+) were generated using the QuikChange method with specific primers (Table S3) as detailed in the manufacturer's protocol (Stratagene, La Jolla, CA). Successful mutations were verified by DNA sequencing. Subconfluent cell cultures were transiently transfected with the respective expression plasmids using the polyethylenimine reagent (Polysciences, Warrington, PA) following the protocol provided by the manufacturer.

### Label-free DMR assay

DMR was recorded as described previously (65, 66) using the Epic System (Corning) together with the Cybi-SELMA semiautomated electronic pipetting system (Analytik Jena AG, Jena, Germany). In brief, 24 h after transfection with the corresponding mutated G protein α subunits, HEK293 cells were counted and seeded at a density of 17,000 cells/well on 384-well fibronectin-coated biosensor plates. On the next day, cells were washed twice with Hanks' buffered salt solution containing 20 mm HEPES (HBSS+HEPES) and incubated for 1 h at 37 °C in the Epic reader. FR and YM were added 1 h before the measurement in HBSS+HEPES. The sensor plate was scanned to record a baseline optical signature (no change in basal DMR), and after agonist addition, DMR changes were monitored for 3,000 s at 37 °C.

### Inositol monophosphate accumulation assay

HTRF®-based IP_1_ accumulation assays (Cisbio, Codolet, France) were performed according to the manufacturer's instructions. In brief, suspensions of 75,000 cells/well (Gα16-expressing cell lines) or 15,000 cells/well (Gαq transfectants) were incubated for 20 min in the presence of LiCl prior to administration of G protein inhibitors alone (Gαq transfectants) or combined treatment with G protein inhibitors and CCh (Gα16 transfectants). G protein inhibitors FR and YM were preincubated with cells for 1 h followed by 30 min of CCh stimulation. Inositol monophosphate accumulation was subsequently measured using a Mithras LB940 multimode plate reader (Berthold Technologies, Bad Wildbad, Germany).

### Western blotting

48 h after transfection, cells were washed twice with PBS and then lysed in ice-cold lysis buffer (25 mm Tris, pH 7.4, 150 mm NaCl, 1 mm EDTA, 1% Triton X-100, 1% IGEPAL) at 4 °C. Lysates were rotated for 20 min at 4 °C and centrifuged at 15,000 × *g* at 4 °C for 10 min. To determine the protein concentration, the Pierce BCA Protein Assay (Thermo Scientific, Waltham, MA) was used following the manufacturer's instructions. Lysates (15 μg of protein) were separated by 10% SDS-PAGE and transferred to nitrocellulose membrane (Hybond^TM^-C Extra from GE Healthcare) by electroblotting. Membranes were shortly washed with PBS containing 0.1% Tween and then blocked with Roti-Block (1×; Carl Roth, Karlsruhe, Germany) for 1 h at room temperature. Afterward, membranes were incubated overnight at 4 °C in Roti-Block containing antibodies specific for Gα16 (1:1,000) or the HA tag (1:1,000). After washing three times with PBS containing 0.1% Tween, membranes were incubated for 1 h at room temperature with a HRP-conjugated secondary goat anti-mouse IgG antibody (1:10,000) in Roti-Block. For signal development of the immunoreactive proteins, the Amersham Biosciences ECL Prime Western blotting detection reagent (GE Healthcare) was used. To normalize for equal loading and protein transfer, membranes were stripped, reprobed with an antibody against β-actin (1:1,000–1:2,500), and visualized after incubation with an HRP-conjugated secondary goat anti-rabbit IgG antibody. Quantification of the immunoreactive bands was carried out by densitometry using ImageJ 1.52a (67) (National Institutes of Health).

### Molecular modeling and structural analysis

All structural analyses are based on the Gq–YM crystal structure (Protein Data Bank (PDB) code 3AH8). The Gα16–FR homology model was constructed with Modeller 9.19 (University of California, San Francisco, CA) using the above-mentioned Gαq–YM crystal structure as template. To adhere as much as possible to the very closely related template structure, the “very fast” keyword was utilized to output the initial model, retaining the copied coordinates for all conserved residue positions. The YM inhibitor from the Gαq template was included in the Gα16 model, and the propionyl plus isopropyl substituents of FR were manually added to the inhibitor in PyMOL 2.0.6 (Schrödinger, New York, NY), ensuring a local minimum “staggered” conformation with the least number of steric clashes. Additionally, the mutations in the G16^FIVE^ mutant were introduced using the mutagenesis wizard in PyMOL, selecting the side-chain rotamer with the highest probability, resemblance to the rotamer of the Gq–YM structure, and few steric clashes with neighboring residues.

### Data analysis

All data were analyzed using GraphPad Prism 8.0.0 (GraphPad Inc., La Jolla, CA). Quantification of DMR signals was performed by calculation of the maximum responses. Data points from concentration-response or inhibition curves of individual functional experiments were fitted to the following four-parameter logistic function.

(Eq. 1) $$Y=\text{Bottom}+\frac{\left(\text{Top}-\text{Bottom}\right)}{1+10^{\left({\text{logEC}}_{50}-x\right)\text{slope}}}$$

Concentration-effect curves represented in figures were normalized by setting each experimental maximal effect as 100% response. All data are expressed as mean + or ± S.E. of at least three independent experiments performed in technical triplicates. Concentration-inhibition curves that did not reach the bottom plateau were constrained to plateau at zero, assuming full inhibition.

## Author contributions

D. M., D. E. G., H. B.-O., G. M. K., and E. K. supervision; D. M., J. P., S. A., K. H., F. E., R. R., M. C., W. H., and D. T. investigation; D. M., J. P., and K. H. visualization; K. H. and E. K. writing-review and editing; R. R., M. C., W. H., H. Z., K. S., G. M. K., and A. I. resources; D. E. G. and E. K. funding acquisition; J. G. and E. K. conceptualization; E. K. writing-original draft; E. K. project administration.

## Supplementary Material

Supporting Information

## Footnotes

6 The abbreviations used are:

GPCR: G protein–coupled receptor
DMR: dynamic mass redistribution
YM: YM-254890
FR: FR900359
vdW: van der Waals
CCh: carbachol
IP_1_: inositol monophosphate
HA: hemagglutinin
HRP: horseradish peroxidase
HBSS: Hanks' buffered salt solution.
