# Supplementary material for: Rational design of a heterotrimeric G protein α subunit with artificial inhibitor sensitivity
Source: J Biol Chem. 2019 Feb 11;294(15):5747–58. doi: 10.1074/jbc.RA118.007250 (PMC6463727; doi:10.1074/jbc.RA118.007250)
Supplement: Supporting Information [file supp_294_15_5747__index.html]

Rational design of a heterotrimeric G protein α subunit with artificial inhibitor sensitivity — Transfer of FR900359 and YM-254890 sites from Gαq to Gα16 — Rational design of a heterotrimeric G protein α subunit with artificial inhibitor sensitivity — Transfer of FR900359 and YM-254890 sites from Gαq to Gα16 — Supporting Information 

# Rational design of a heterotrimeric G protein α subunit with artificial inhibitor sensitivity

## Supporting Information

- Supporting Information (to be published online) - Supplementary Figures and Tables
