# Supplementary material for: Rational design of a heterotrimeric G protein α subunit with artificial inhibitor sensitivity
Source: J Biol Chem. 2019 Feb 11;294(15):5747–58. doi: 10.1074/jbc.RA118.007250 (PMC6463727; doi:10.1074/jbc.RA118.007250)
Supplement: Supporting Information [file supp_RA118.007250_142706_1_supp_261661_pmrryy.pdf]

SUPPORTING INFORMATION

Rational design of a heterotrimeric G protein α subunit with artificial inhibitor sensitivity

Davide Malfacini[1,&], Julian Patt[1,&], Suvi Annala[1], Kasper Harpsøe[2], Funda Eryilmaz[1], Raphael Reher[3,#], Max Crüsemann[3], Wiebke Hanke[3], Hang Zhang[2,θ], Daniel Tietze[4], David E. Gloriam[2], Hans Bräuner-Osborne[2], Kristian Strømgaard[2], Gabriele M. König[3], Asuka Inoue[5], Jesus Gomeza[1], Evi Kostenis[1*]

[1]Molecular, Cellular and Pharmacobiology Section, Institute for Pharmaceutical Biology, University of Bonn, Nussallee 6, 53115, Bonn, Germany. [2]Department of Drug Design and Pharmacology, University of Copenhagen, Universitetsparken 2, 2100, Copenhagen, Denmark. [3]Institute for Pharmaceutical Biology, University of Bonn, Nussallee 6, 53115, Bonn, Germany. [4]Eduard-Zintl-Institute of Inorganic and Physical Chemistry, Technische Universität Darmstadt, 64287 Darmstadt, Germany. [5]Graduate School of Pharmaceutical Sciences, Tohoku University, 6-3, Aoba, Aramaki, Aoba-ku, Sendai, Miyagi 980-8578, Japan.

Running title: *Transfer of FR900359 and YM-254890 sites from Gαq to Gα16*

& These authors contributed equally to this work.

# Present address: Scripps Institution of Oceanography, Center for Marine Biology & Biomedicine, UC San Diego, CA 92093, La Jolla, USA.

θ Present address: Key Laboratory of Advanced Drug, Preparation Technologies, Ministry of Education, Co-innovation Center of Henan Province for New Drug R & D and Preclinical Safety, and School of Pharmaceutical Sciences, Zhengzhou University, 100 Kexue Avenue, Zhengzhou, Henan 450001, China.

* To whom correspondence should be addressed: Prof. Evi Kostenis; Molecular, Cellular and Pharmacobiology Section, Institute for Pharmaceutical Biology, University of Bonn, Nussallee 6, 53115, Bonn, Germany. kostenis@uni-bonn.de.

Table of Contents

Figure S1	**CRISPR-Cas9 HEK293 Gαq/Gα11-null cells do not respond to canonical Gq stimuli.**	page S-3
Figure S2	**FR does not alter whole cell activation profiles in the genetic absence of Gαq and Gα11.**	page S-3
Figure S3	**FR but not YM attenuates G16-mediated inositol phosphate formation.**	page S-4
Figure S4	**The quintuple loss-of-function mutant Gq^{FIVE} recapitulates the pharmacological profiles of FR and YM on native G16.**	page S-4
Figure S5	**Functional phenotypes of mutant Gαq proteins.**	page S-5
Figure S6	**Maximal signaling amplitudes of Gαq mutant proteins correlate with their cellular abundance.**	page S-6
Figure S7	**Functional phenotypes of mutant Gα16 proteins.**	page S-7
Figure S8	**Maximal signaling amplitudes of Gα16 mutant proteins correlate with their cellular abundance.**	page S-8
Figure S9	**Quantitative reconstruction of functional FR and YM sites within Gα16.**	page S-9
Figure S10	**Gα16 Phe78 mutants are functional and detected by immunoblot with their expected band size.**	page S-10
Figure S11	**Potency profiles of FR and YM on single *vs.* multiple mutants indicate interdependence of manifold simultaneous interactions between inhibitors and their target proteins.**	page S-11
Table S1	**Quantification of FR, YM and CCh activities at wild type and mutant Gαq proteins in Gαq/Gα11-null cells.**	page S-12
Table S2	**Quantification of FR, YM and CCh activities at wild type and mutant Gα16 proteins in Gαq/Gα11-null cells.**	page S-13
Table S3	**Primers for site-directed mutagenesis.**	page S-14

Figure S1.

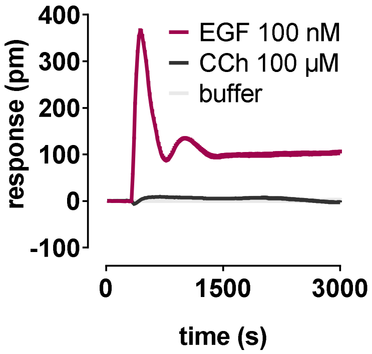

CRISPR-Cas9 HEK293 Gαq/Gα11-null cells do not respond to canonical Gq stimuli.
DMR analysis of whole cell responses evoked by epidermal growth factor (EGF) and CCh at the indicated concentrations in CRISPR-Cas9 genome-edited HEK293 cells that lack functional alleles for Gαq and Gα11. Shown are real-time measurements (mean + s.e.m., technical triplicates) representative of three such experiments.

Figure S2.

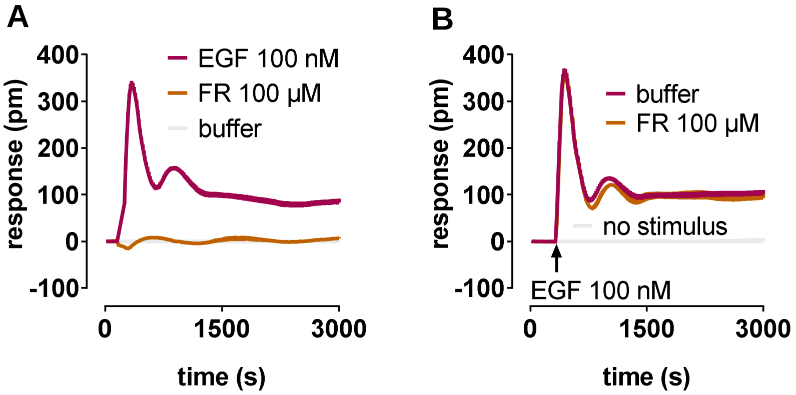

FR does not alter whole cell activation profiles in the genetic absence of Gαq and Gα11.
(A) CRISPR-Cas9 Gαq/Gα11-null cells respond to epidermal growth factor (EGF) but not to FR with robust alteration of DMR profiles. (B) FR does not block EGF-mediated whole cell responses. Data shown are representative real-time recordings (mean + s.e.m., technical triplicates) of three independent experiments.

Figure S3.

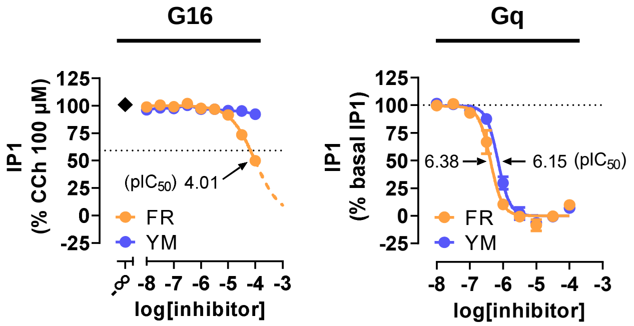

FR but not YM attenuates G16-mediated inositol phosphate formation.
Accumulation of inositol phosphates in CRISPR-Cas9 Gαq/Gα11-null cells transfected to express Gα16 or Gαq. Cells were pretreated with varying concentrations of FR or YM prior to stimulation with 100 µM CCh (Gα16 transfectants) or vehicle control (Gαq transfectants). Data represent means ± s.e.m from n= 5 independent experiments each performed as technical triplicates. The stippled line indicates the magnitude of basal IP1 production in the absence of CCh. CCh efficacy at 100 µM is shown as black diamond.

Figure S4.

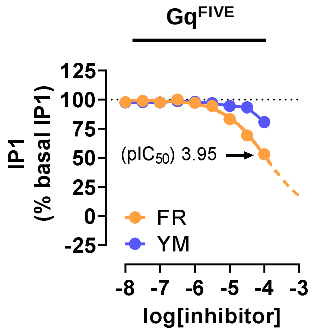

The quintuple loss-of-function mutant Gq^{FIVE} recapitulates the pharmacological profiles of FR and YM on native G16.
Accumulation of inositol phosphates in CRISPR-Cas9 Gαq/Gα11-null cells transfected to express Gαq^{FIVE}. Cell-intrinsic IP1 accumulation was quantified in the absence and presence of varying concentrations of FR or YM. Data represent means ± s.e.m from n= 5 independent experiments each performed as technical triplicates. The stippled line indicates the magnitude of basal IP1 production in the absence of CCh.

Figure S5.

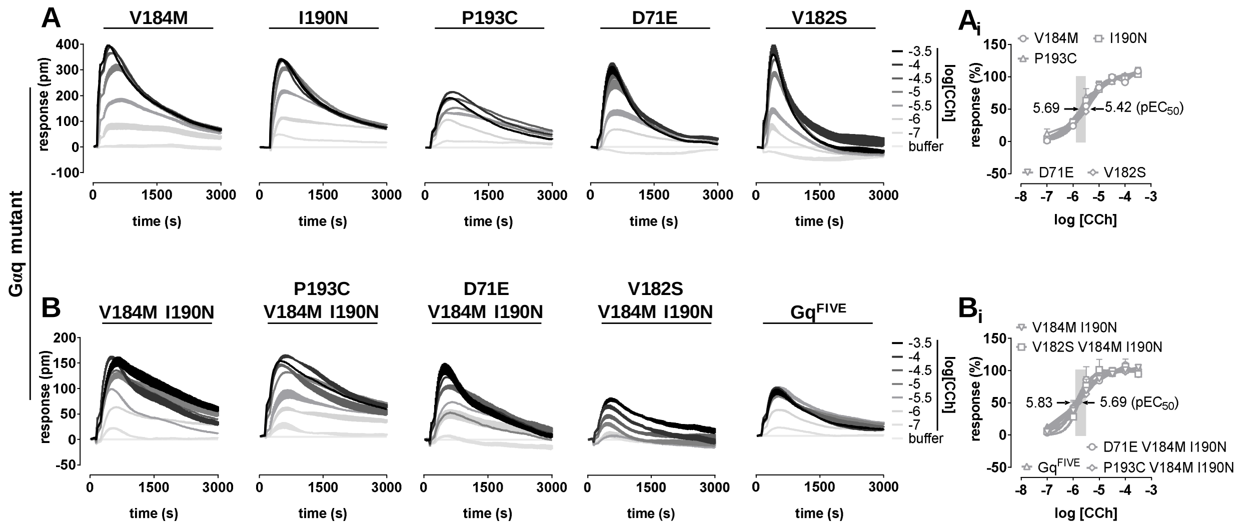

Functional phenotypes of mutant Gαq proteins.

(A, B) DMR analysis of G protein-dependent whole cell responses evoked by the indicated concentrations of CCh in CRISPR-Cas9 Gαq/Gα11-null cells enriched by transfection with mutant Gαq subunits. Whole cell activation profiles of all Gαq mutants were in a similar range as compared with the respective wild type control (grey area indicates deviation by a factor of 1.5 from the EC_{50} values obtained for Gαq wild type). Data are shown as representative real-time measurements (A, B, technical triplicates) with corresponding concentration-effect curves (A_i, B_i, means ± s.e.m. from at least 3 independent biological replicates).

Figure S6.

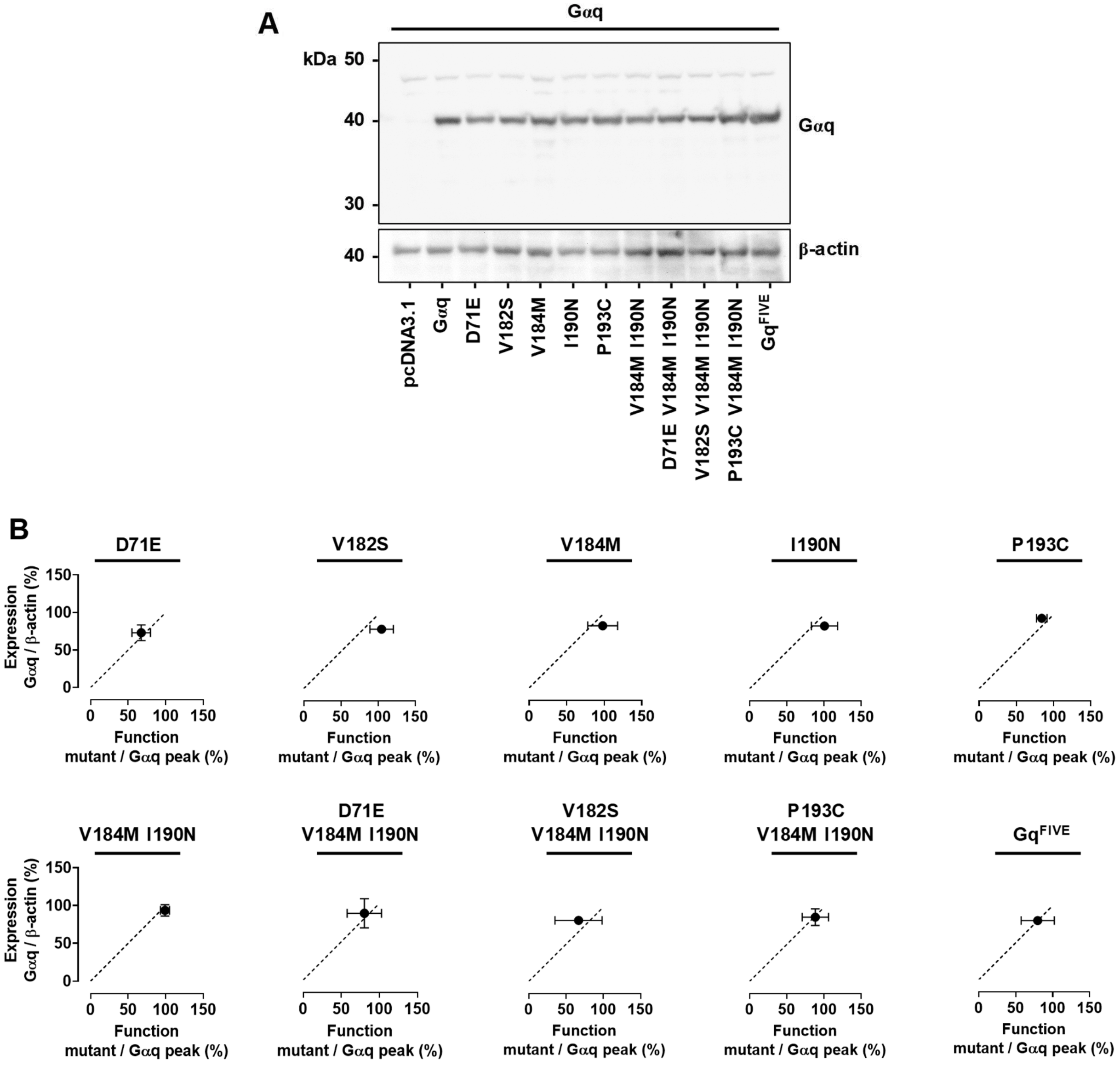

Maximal signaling amplitudes of Gαq mutant proteins correlate with their cellular abundance.
(A) representative western blot showing expression levels of Gαq wild type and mutant proteins detected in cellular lysates that were collected from CRISPR-Cas9 Gαq/Gαq11-null cells transiently expressing the indicated constructs. β-actin was used as loading control. (B) Correlation of α-subunit function (normalized maximal DMR amplitude at t ~ 500 s in response to a saturating CCh concentration of 300 µM) with relative cellular abundance is shown for each mutant relative to Gαq wild type control. The stippled line indicates a positive linear relationship between function and expression with margins set by vector control and Gαq wild type which are arbitrarily denoted as 0 and 100, respectively. Data are means ± s.e.m. of 3 independent experiments.

Figure S7.

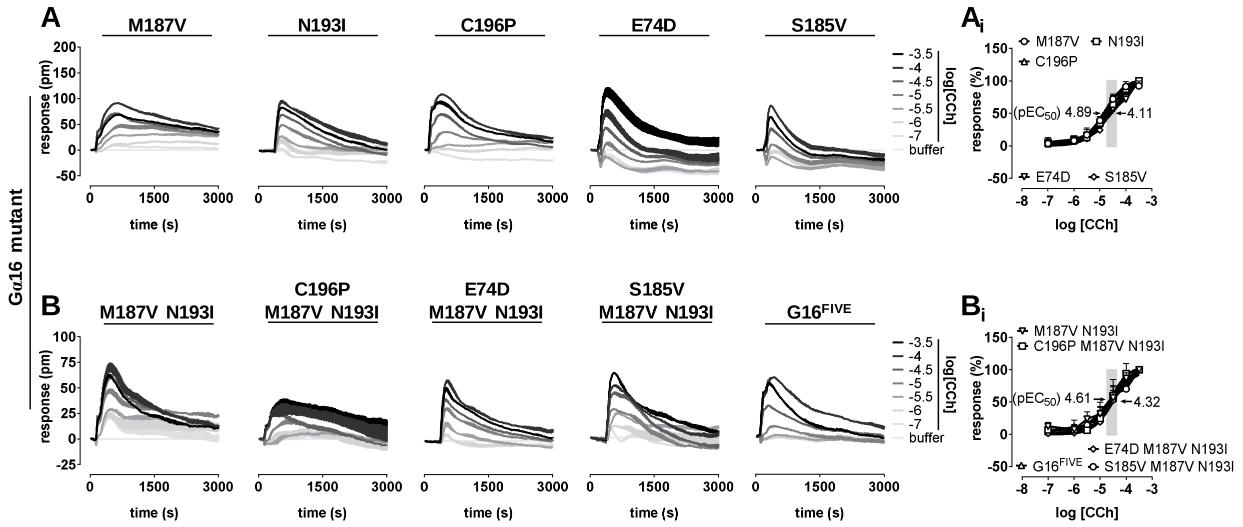

Functional phenotypes of mutant Gα16 proteins.

(A, B) DMR analysis of G protein-dependent whole cell responses evoked by the indicated concentrations of CCh in CRISPR-Cas9 Gαq/Gα11-null cells enriched by transfection with mutant Gα16 subunits. Whole cell activation profiles of all Gα16 mutants were in a similar range as compared with the respective wild type control (grey area indicates deviation by a factor of 1.5 from the EC_{50} value obtained for Gα16 wild type). Data are shown as representative real-time measurements (A, B, technical triplicates) with corresponding concentration-effect curves (A_i, B_i, means ± s.e.m. from at least 3 independent biological replicates).

Figure S8.

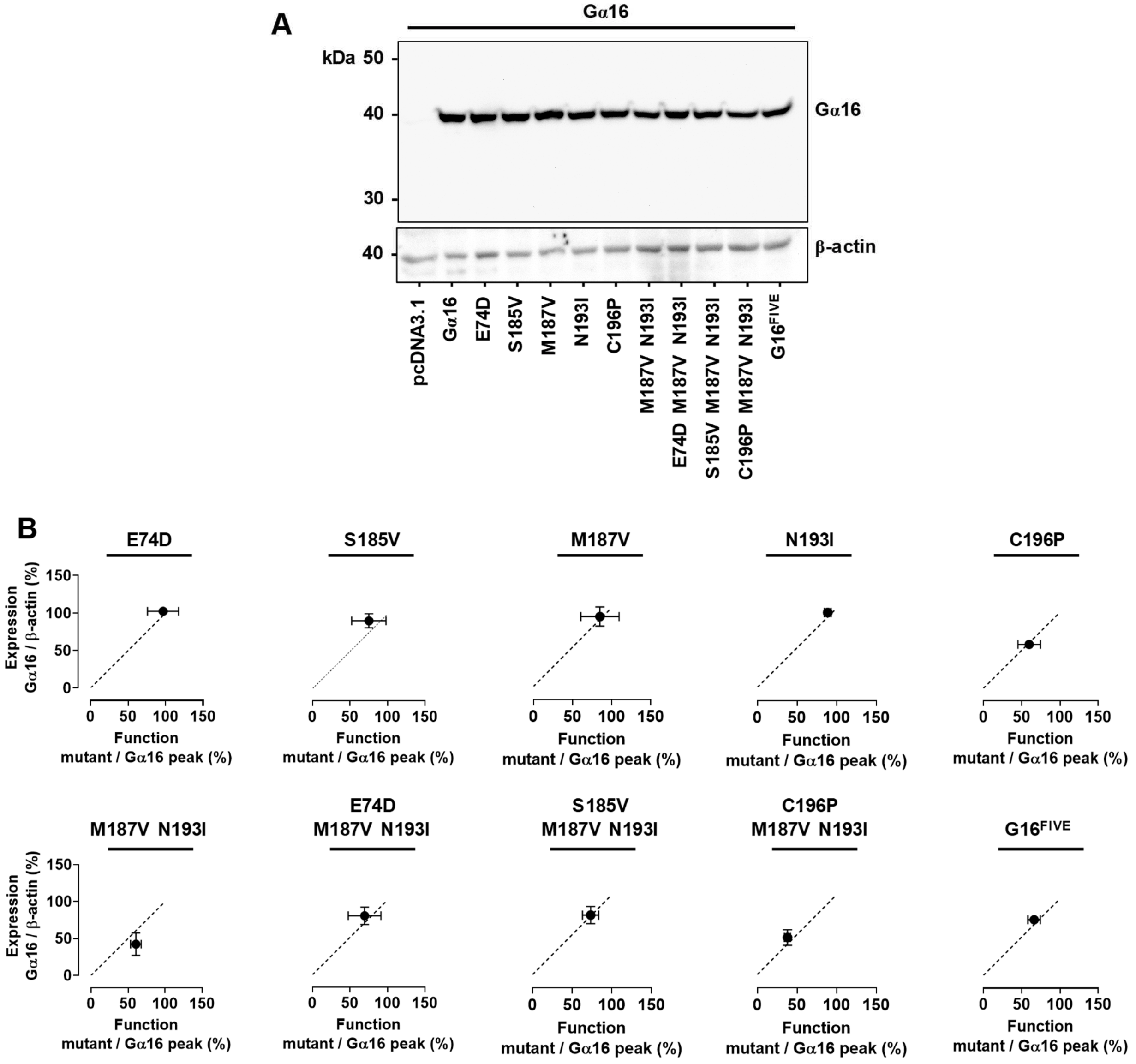

Maximal signaling amplitudes of Gα16 mutant proteins correlate with their cellular abundance.
(A) representative western blot showing expression levels of Gα16 wild type and mutant proteins detected in cellular lysates that were collected from CRISPR-Cas9 Gαq/Gαq11-null cells transiently expressing the indicated constructs. β-actin was used as loading control. (B) Correlation of α-subunit function (normalized maximal DMR amplitude at t ~ 500 s in response to a saturating CCh concentration of 300 µM) with relative cellular abundance is shown for each mutant relative to Gα16 wild type control. The stippled line indicates a positive linear relationship between function and expression with margins set by vector control and Gα16 wild type which are arbitrarily denoted as 0 and 100, respectively. Data are means ± s.e.m. of 3 independent experiments.

Figure S9.

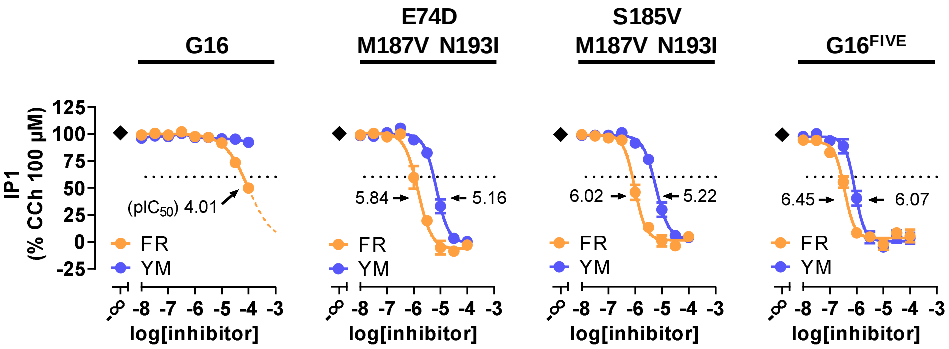

Quantitative reconstruction of functional FR and YM sites within Gα16.
Accumulation of inositol phosphates in CRISPR-Cas9 Gαq/Gα11-null cells transfected to express Gα16 wild type or the indicated triple or quintuple mutants. Cells were pretreated with varying concentrations of FR or YM prior to stimulation with 100 µM CCh. Data represent means ± s.e.m from n= 5 independent experiments each performed as technical triplicates. The stippled line indicates the magnitude of basal IP1 production in the absence of CCh. CCh efficacy at 100 µM is shown as black diamond.

Figure S10.

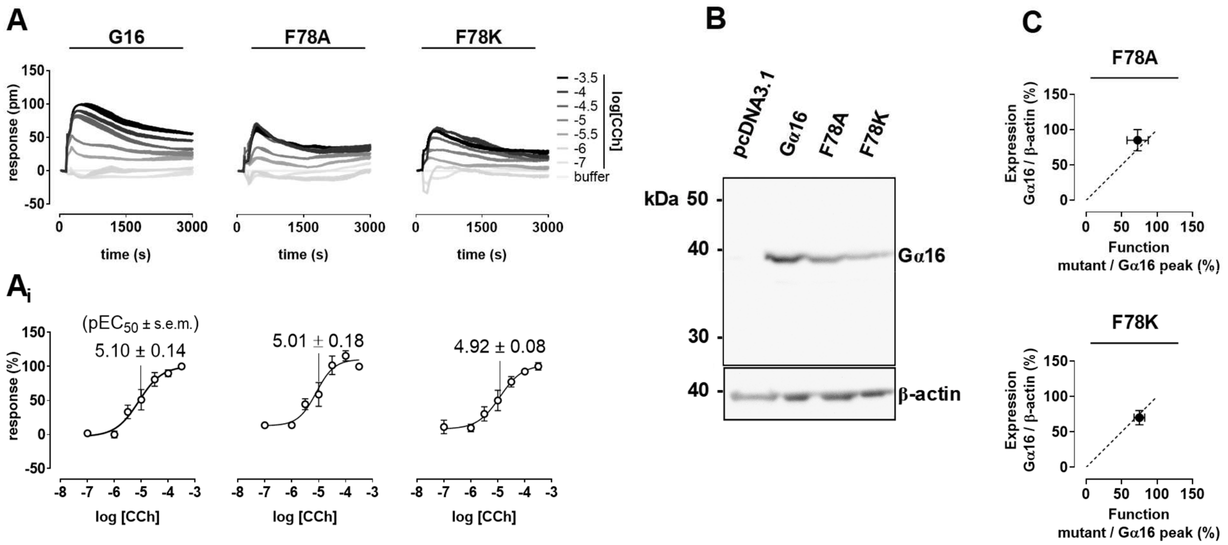

Gα16 Phe78 mutants are functional and detected by immunoblot with their expected band size.
(A) DMR profiling of G protein-dependent whole cell responses upon stimulation by CCh in CRISPR-Cas9 Gαq/Gα11-null cells transiently expressing the indicated proteins. Data shown are representative real-time recordings (A) and corresponding concentration-effect curves (A_i, means ± s.e.m., n= 3). (B) Representative immunoblot showing cellular abundance of Gα16 wild type and missense mutants in lysates collected from CRISPR-Cas9 Gαq/Gα11-null cells ectopically enriched with the indicated constructs after transient transfection. β-actin was used as loading control, two additional blots from independent transfections gave similar results. (C) Correlation of α-subunit function (normalized maximal DMR amplitude at t ~ 500 s in response to a saturating CCh concentration of 300 µM) with relative cellular abundance of the indicated proteins is shown. Data represent means ± s.e.m. of 3 independent experiments.

Figure S11.

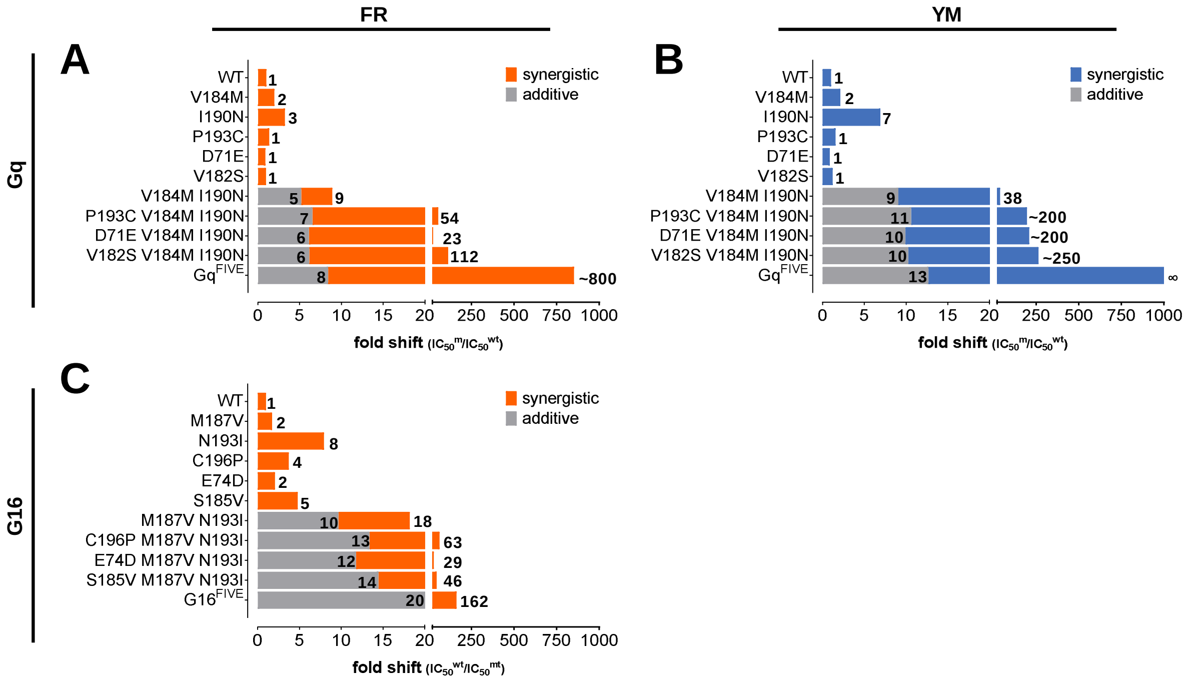

Potency profiles of FR and YM on single *vs.* multiple mutants indicate interdependence of manifold simultaneous interactions between inhibitors and their target proteins.

(A-C) Ratios of half maximal inhibitory concentrations (IC_{50}) between FR (A, C) or YM effects (B) in mutated and wild type Gαq and Gα16 proteins. The extent of synergism is colored in orange (for FR) or blue (for YM), and was directly calculated from experimental data (**Tables S1** and **S2**). Additive effects (grey portion of the bars) are the sum of shifts corresponding to theoretical loss (A, B) or gain of function (C) for the inhibitors on the respective mutant proteins. Combined effects on multiple mutants were always greater that those predicted by their individual potencies for both FR and YM, attesting to multiple simultaneous interactions between FR or YM and their protein targets. Inactivity of YM on G16 did not allow quantification of additive versus synergistic effects for the gain of function mutants. Inactivity of YM on Gq^{FIVE} (B) is denoted by an infinity symbol.

Table S1.

**Gαq isoform**	**Secondary structure features**	**FR** pIC50 ± s.e.m.	**YM** pIC50 ± s.e.m.	n	**CCh** pEC50 ± s.e.m.	n
WT	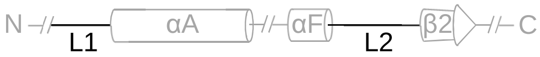	6.28 ± 0.04	6.27± 0.03	8	5.71 ± 0.06	4
V184M	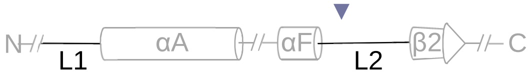	5.99 ± 0.02	5.95 ± 0.02	3	5.55 ± 0.07	3
I190N	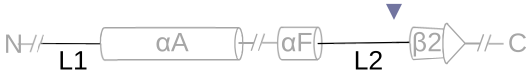	5.77 ± 0.03	5.44 ± 0.04	8	5.64 ± 0.05	3
P193C	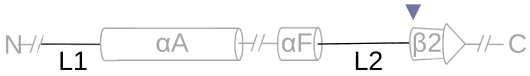	6.15 ± 0.09	6.09 ± 0.07	4	5.65 ± 0.12	3
D71E	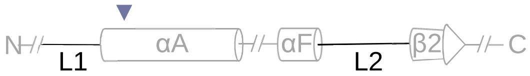	6.32 ± 0.08	6.34 ± 0.05	3	5.69 ± 0.08	3
V182S	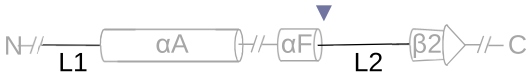	6.30 ± 0.04	6.20 ± 0.04	4	5.42 ± 0.08	3
V184M I190N	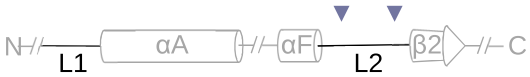	5.33 ± 0.05	4.70 ± 0.10	7(3)	5.69 ± 0.05	3
P193C V184M I190N	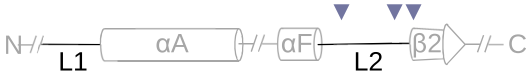	4.55 ± 0.06	3.99 ± 0.08	7(3)	5.77 ± 0.07	3
D71E V184M I190N	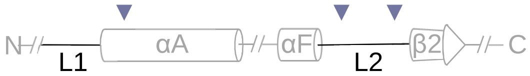	4.91 ± 0.09	3.96 ± 0.08	5(3)	5.82 ± 0.05	3
V182S V184M I190N	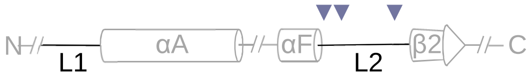	4.23 ± 0.09	3.86 ± 0.13	5(3)	5.70 ± 0.11	3
Gq^{FIVE} (D71E V182S V184M I190N P193C)	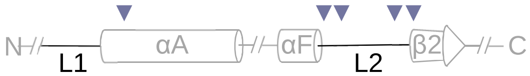	3.95± 0.08	< 3	7(3)	5.83 ± 0.09	3

Quantification of FR, YM and CCh activities at wild type and mutant Gαq proteins in Gαq/Gα11-null cells. Listed are Gαq wild type and the entire set of loss of function mutants including their diagrammatic representation; arrowheads indicate the location of each mutation within the secondary structure elements. pIC_{50} (FR, YM) and pEC_{50} (CCh) values were determined by nonlinear regression on concentration-effect data. Each value is the average (± s.e.m.) of 'n' independent biological replicates performed as technical triplicates. FR and YM replicates using high (32-100 µM) concentrations are indicated in brackets.

Table S2.

**Gα16 isoform**	**Secondary structure features**	**FR** pIC50 ± s.e.m.	**YM** pIC50 ± s.e.m.	n	**CCh** pEC50 ± s.e.m.	n
WT	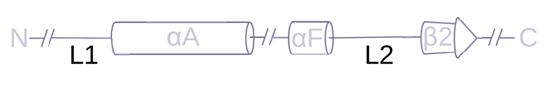	4.05 ± 0.03	< 3	17(9)	4.56 ± 0.11	4
M187V	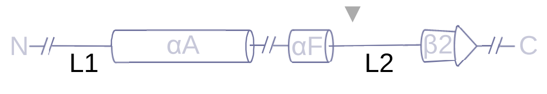	4.29 ± 0.08	< 3	7(3)	4.89 ± 0.09	3
N193I	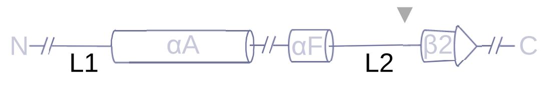	4.95 ± 0.06	3.87 ± 0.09	10(6)	4.65 ± 0.12	3
C196P	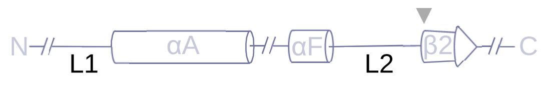	4.62 ± 0.07	< 3	6(4)	4.78 ± 0.08	3
E74D	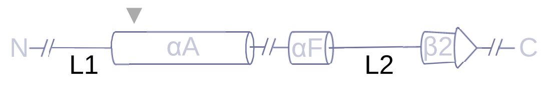	4.37 ± 0.09	< 3	7(3)	4.11 ± 0.60	3
S185V	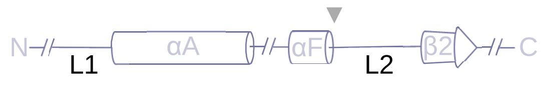	4.73 ± 0.05	~ 3.5	7(3)	4.49 ± 0.24	3
M187V N193I	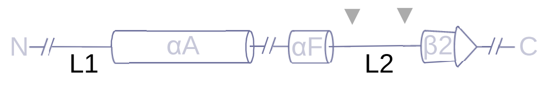	5.31 ± 0.05	4.48 ± 0.09	7(3)	4.39 ± 0.37	3
C196P M187V N193I	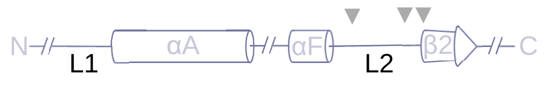	5.85 ± 0.09	5.38 ± 0.11	6(3)	4.61 ± 0.18	3
E74D M187V N193I	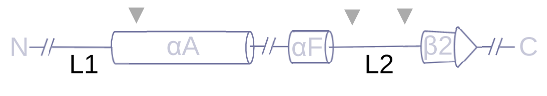	5.61 ± 0.05	5.06 ± 0.08	6(3)	4.54 ± 0.11	3
S185V M187V N193I	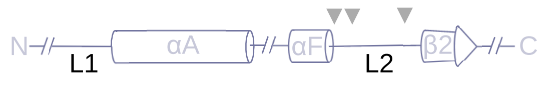	5.71 ± 0.05	4.96 ± 0.09	6(3)	4.32 ± 0.59	3
G16^{FIVE} (E74D S185V M187V N193I C196P)	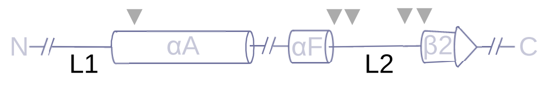	6.26 ± 0.07	6.15 ± 0.09	5	4.47 ± 0.31	3

Quantification of FR, YM and CCh activities at wild type and mutant Gα16 proteins in Gαq/Gα11-null cells. Listed are Gα16 wild type and the entire set of gain of function mutants including their diagrammatic representation; arrowheads indicate the location of each mutation within the secondary structure elements. pIC$_{50}$ (FR, YM) and pEC$_{50}$ (CCh) values were determined by nonlinear regression on concentration-effect data. Each value is the average (± s.e.m.) of 'n' independent biological replicates performed as technical triplicates. FR and YM replicates using high (32-100 µM) concentrations are indicated in brackets.

Table S3.

Mutant	Forward primer (5′–3′)	Reverse primer (5′–3′)
Gq D71E	CTGACGAAGAGAAGCGCGG CTTCACCAAGC	GCTTGGTGAAGCCGCGCTTCTC TTCGTCAG
Gq V182S	GTGCTTAGAAGTCGAGTCCC CACTACAGGGATC	GATCCCTGTAGTGGGACTCG ACTTCTAAGCAC
Gq V184M	GCTTAGAGTTCGAATGCCCA CTACAGGGATC	GTAGTGGGCATTCGAACTCTA AGCACGTCTTGTTG
Gq I190N	CAGGATCAACGAATACCCCT TTGACTTACAAAG	CAAAGGGGTATTCGTTGATCC CTGTAGTGGG
Gq P193C	CATCGAATACTGCTTTGACTT ACAAAGTGTC	CACTTTGTAAGTCAAAGCAGT ATTCGATG
Gq V182S V184M I190N	CACAACAAGACGTGCTTAGA AGTCGAATGCCC	GGGCATTCGACTTCTAAGCAC GTCTTGTTGTG
Gq P193C V184M I190N	AACGAATACTGCTTTGACTT ACAAAGTG	CACTTTGTAAGTCAAAGCAGT ATTCGTT
G16 E74D	CTCGGAGGAGGACCGCAAG GGCTTCCGGCCCCTG	CAGGGGCCGGAAGCCCTTGCG GTCCTCCTCCGAG
G16 F78A	GGAGCGCAAGGGCGCGCGG CCCCTGGTCTAC	GTAGACCAGGGGCCGCGCGCC CTTGCGCTCC
G16 F78K	GGAGCGCAAGGGCAAGCGG CCCCTGGTCTAC	GTAGACCAGGGGCCGCTTGCC CTTGCGCTCC
G16 S185V	TGCTTCGCGTGCGCATGCCC ACCACTG	GGCATGCGCACGCGGAGCACG TCCTGA
G16 M187V	CGCAGCCGCGTACCCACTAC TGGCATCAACG	CAGTGGTGGGTACGCGGCTTC GGAGCACGTCC
G16 N193I	CCACTGGCATCATCGAGTAC TGCTTCTCCGTGC	GCACGGAGAAGCAGTACTCGA TGATGCCAGTGG
G16 C196P	CAACGAGTACCCCTTCTCCG TGCAGAAAACCAAC	GTTGGTTTTCTGCACGGAGAA GGGGTACTCGTTG
G16 S185V M187V N193I	GACGTGCTCCGTGTCCGCGT ACCCACC	GGTGGGTACGCGGACACGGAG CACGTC
G16 C196P M187V N193I	CACTGGCATCATCGAGTACC CCTTCTCCGTGC	GCACGGAGAAGGGGTACTCGA TGATGCCAGTG

Primers for site-directed mutagenesis. QuikChange forward and reverse primers employed to generate the indicated Gαq and Gα16 loss or gain of function constructs, respectively, are listed. Mutant constructs not mentioned therein were created in multiple steps from previously generated scaffolds.
